# 469. Outcomes of COVID-19 among hospitalized patients under Rituximab: the experience of a Portuguese hospital

**DOI:** 10.1093/ofid/ofad500.539

**Published:** 2023-11-27

**Authors:** Maria João M S Gonçalves, Eduarda Pena, Frederico Duarte, Susana Oliveira, Clara Batista, Mário Guimarães, Ricardo Correia Abreu, Isabel Neves, Sofia Jordão

**Affiliations:** Hospital Pedro Hispano - Unidade Local Saúde Matosinhos, Matosinhos, Porto, Portugal; Hospital Pedro Hispano - Unidade Local Saúde Matosinhos, Matosinhos, Porto, Portugal; Hospital Pedro Hispano - Unidade Local Saúde Matosinhos, Matosinhos, Porto, Portugal; Hospital Pedro Hispano - Unidade Local Saúde Matosinhos, Matosinhos, Porto, Portugal; Hospital Pedro Hispano - Unidade Local de Saúde de Matosinhos, Matosinhos, Porto, Portugal; Hospital Pedro Hispano - Unidade Local Saúde Matosinhos, Matosinhos, Porto, Portugal; Hospital Pedro Hispano - Unidade Local Saúde Matosinhos, Matosinhos, Porto, Portugal; Hospital Pedro Hispano - Unidade Local de Saúde de Matosinhos, Matosinhos, Porto, Portugal; Hospital Pedro Hispano - Unidade Local Saúde Matosinhos, Matosinhos, Porto, Portugal

## Abstract

**Background:**

Rituximab (RTX) is a monoclonal antibody commonly used for various hematological and autoimmune disorders. B-cell–depleting therapies also blunt humoral responses to SARS-CoV-2 vaccines. Whether this carries an increased risk of hospitalization or death from COVID-19 is unclear.

**Methods:**

We conducted a retrospective study of hospitalized patients with COVID-19 who received RTX prior to hospitalization (maximum 18 months before). We analysed their outcomes, including length of stay, antiviral treatment, inflamatory markers and mortality.

**Results:**

A total of 43 hospitalized COVID-19 patients who received RTX were included in the study (from April 2020 to April 2023) . The median age was 67 years (range, 25-88), and 58% were female. The average length of stay was 28 days (range, 1-29). Upon admission, average value of severity predictor markers: CRP 94.6 mg/L, procalcitonine 0.66 ng/mL, lactate dehydrogenase (LDH) 383.5 U/L, D-dimers 1530.3 ng/mL. Treatment was performed in 44% with dexamethasone and in 48% with remdesivir. The mortality rate was 42%.

**Conclusion:**

In our study, previous treatment with rituximab was associated with high severity predictor markers and serious outcomes of COVID-19.

Patients and healthcare professionals should be aware of the risk associated with COVID-19 in this population. Clinicians must choose carefully patients for anti-CD20 treatments. Preventive measures, active and passive immunization, as well as early antivirals therapy may decrease COVID-19 severity.

**Disclosures:**

**All Authors**: No reported disclosures

